# Correction: Stereomutation and chiroptical bias in the kinetically controlled supramolecular polymerization of cyano-luminogens

**DOI:** 10.1039/d2sc90230c

**Published:** 2022-11-14

**Authors:** Lucía López-Gandul, Cristina Naranjo, Cecilia Sánchez, Rafael Rodríguez, Fátima Aparicio, Rafael Gómez, Jeanne Crassous, Luis Sánchez

**Affiliations:** Departamento de Química Orgánica, Facultad; de Ciencias Químicas, Universidad Complutense de Madrid Madrid 28040 Spain lusamar@ucm.es; Univ Rennes, CNRS, ISCR (Institut des Sciences Chimiques de Rennes) – UMR 6226 Rennes F-35000 France jeanne.crassous@univ-rennes1.fr

## Abstract

Correction for ‘Stereomutation and chiroptical bias in the kinetically controlled supramolecular polymerization of cyano-luminogens’ by Lucía López-Gandul *et al.*, *Chem. Sci.*, 2022, **13**, 11577–11584, https://doi.org/10.1039/D2SC03449B.

In the original manuscript, Fátima Aparicio was accidentally omitted from the author list. Fátima Aparicio contributed to the work with the synthesis of some of the reported compounds. The corrected list above demonstrates how the author names should be displayed for this article.

The Royal Society of Chemistry apologises for these errors and any consequent inconvenience to authors and readers.

## Supplementary Material

